# Joint association of sleep quality and physical activity with hypertension: a cross-sectional population study in agricultural workers

**DOI:** 10.3389/fcvm.2025.1618094

**Published:** 2025-08-11

**Authors:** Yu-Fei Chen, Qian Zhao, Xieyire Hamulati, Liting Cai, Xinyu Qiu, Jiamule Maimaitiyiming, Fen Liu, Xiao-Mei Li, Yi-Ning Yang

**Affiliations:** ^1^ Department of Coronary Heart Disease, The First Affiliated Hospital of Xinjiang Medical University, Urumqi, Xinjiang, China; ^2^ School of Public Health, Xinjiang Medical University, Urumqi, Xinjiang, China; ^3^ School of Health Management, Xinjiang Medical University, Urumqi, Xinjiang, China; ^4^ Xinjiang Key Laboratory of Cardiovascular Disease, Clinical Medical Research Institute, The First Affiliated Hospital of Xinjiang Medical University, Urumqi, Xinjiang, China; ^5^ Department of Cardiology, People’s Hospital of Xinjiang Uygur Autonomous Region, Urumqi, Xinjiang, China

**Keywords:** hypertension, association, sleep quality, physical activity, agricultural workers

## Abstract

**Objective:**

The study explores the prevalence of hypertension and evaluates the joint association of sleep quality and physical activity (PA) levels in influencing hypertension among the Kazakh agricultural workers in Xinjiang.

**Methods:**

In this chronic disease study conducted in Xinjiang, participants were selected from Habahe County, a predominantly Kazakh region, between October and November 2023. Restricted cubic spline (RCS) analysis and multivariate logistic regression models were used to evaluate the associations between sleep quality, PA levels, and the prevalence of hypertension.

**Results:**

In this study of 2,872 participants, the median age was 49 (42–57) years. Among participants, 1,253 (43.63%) were male. The study resulting in a hypertension prevalence of 54.94%. RCS regression showed a linear association between sleep quality and hypertension prevalence in participants (*P* overall = 0.007, *P* nonlinear = 0.214), and the association between PA level and hypertension prevalence was also statistically significant (*P* overall = 0.022). As PA level increased, hypertension prevalence gradually declined but stabilized at higher level. In a multivariate regression analysis adjusting for potential confounders, poorer sleep quality was associated with an increased prevalence of hypertension (OR = 1.33, 95% CI: 1.07–1.65, *P* = 0.011; OR = 1.38, 95% CI: 1.12–1.69, *P* = 0.001), while the association between PA level and hypertension prevalence was not statistically significant (*P* > 0.05). Further analysis showed that in the moderate PA level group, good sleep quality was significantly associated with a lower prevalence of hypertension (OR = 0.71, 95% CI: 0.52–0.97, *P* = 0.031). However, in individuals with low (<6,000 METs × min/week) and high (>12,000 METs × min/week) PA levels, the association between sleep quality and hypertension prevalence was not been observed (*P* > 0.05).

**Conclusion:**

The current study showed that PA and sleep quality are associated with the prevalence of hypertension. Among individuals with moderate PA level, healthy sleep quality may have a protective effect against hypertension.

## 1 Introduction

Hypertension remains the foremost modifiable risk factor linked to cardiovascular diseases and premature death globally (1). Over the years, its incidence has escalated, imposing a significant burden on public health (2–4). Persistent high blood pressure induces structural and functional alterations in target organs and significantly increases the risk of severe complications, including myocardial infarction, stroke, and kidney failure (5, 6). Furthermore, hypertension adversely affects general health beyond the cardiovascular system, manifesting in symptoms such as headaches (7). These effects markedly compromise patient quality of life, imposing a significant socioeconomic burden on healthcare systems.

Adequate sleep, balanced diet, and regular exercise are pivotal in the holistic approach to managing hypertension (8–10), as delineated in the new ACC/AHA hypertension guidelines (11). The quality of sleep emerges as a prominent influence cardiovascular health, including the risk of hypertension and other cardiovascular diseases (12, 13). Previous studies have emphasized that maintaining healthy sleep pattern and ensuring adequate sleep duration are crucial for reducing hypertension risk (14–16). Moreover, physical activity (PA), particularly during leisure PA, is widely recognized for its beneficial effects on lowering blood pressure and enhancing heart health (17–19). The combined impact of sleep and PA could potentially synergize to lower hypertension risks through diverse mechanisms (20, 21). While much of the existing research has focused on the separate contributions of sleep quality and PA towards hypertension reduction primarily in urban settings (21–23), the association of sleep quality, PA, and their potential interactions in agricultural workers remains unclear. In this study, we leveraged the 2023 baseline data from the population-based cohort study of chronic diseases in Xinjiang (PCCDX) to examine the association between PA level, sleep quality, and the prevalence of hypertension among agricultural workers.

## 2 Methods

### 2.1 Participants

This population-based, cross-sectional study was conducted between October and November 2023. A random sampling method was used to select two towns in Habahe County, Altay Region, predominantly inhabited by the Kazakh population. Participants from 27 administrative villages within these towns were recruited using convenience sampling. Documented consent was acquired in writing from each participant prior to the study. The study protocol was approved by the Ethics Committee of the First Affiliated Hospital of Xinjiang Medical University (Approval No. K202001-06).

### 2.2 Measures

Before the initiation of the research, 8 researchers were comprehensively trained to harmonize the methodology for data collection. They conducted structured interviews with each participant using a standardized paper questionnaire. This survey was designed to identify and explore potential confounding variables that could affect the relationships among sleep quality, PA levels, and hypertension. Factors such as demographic details, medical history, and related lifestyle factors were meticulously documented to enhance the reliability of the findings.

In this investigation, sleep quality was evaluated through the application of the Pittsburgh Sleep Quality Index (PSQI), which is widely recognized for its effectiveness in assessing sleep patterns (22–24). Scores from the PSQI were segmented into three categories (25): healthy sleep quality (≤5 scores), intermediate sleep quality (5–8 scores), and poor sleep quality (>8 scores). PA levels were evaluated using the modified short-form International Physical Activity Questionnaire (IPAQ) (26). IPAQ is a widely used for assessing PA levels across diverse populations and various types of PA (27–29). The accumulation of weekly PA was quantified in terms of total metabolic equivalent tasks (METs), determined by the product of MET values assigned to different activities and the duration of these activities each week (30). The categorization of PA levels was established as low PA (≤6,000 METs × min/week), moderate PA (6,000–12,000 METs × min/week), and high PA (>12,000 METs × min/week).

This investigation included multiple covariates such as sex, age, educational attainment (below senior high school or above senior high school), marital status (married or other), household annual total income (<60,000 CNY or ≥60,000 CNY), smoking status (“yes” or “no”), alcohol ingestion (“yes” or “no”), body mass index (BMI), and dietary intakes. Current smokers are defined as individuals who reported having smoked at least one cigarette daily for 6 months, or at least 100 cigarettes in their lives. Current drinkers were specified as those who had drunk alcohol at least once a week during the previous 12 months (18). The assessment of dietary intakes involved querying participants regarding how often they consumed prevalent food types over the previous year (categorized as never/occasionally, ≤3 times per month, 1–2 times per week, 3–4 times per week, 5–6 times per week, or daily). The Dietary diversity score (DDS) (31, 32), which is based on the Chinese Dietary Guidelines, considers nine categories of food: vegetables, fruits, legumes, nuts, meats, eggs, fish, tea, milk, and their derivatives. A DDS between 0 and 9 is used, where higher scores reflect a broader variety of diet.

### 2.3 Hypertension

Hypertension was defined based on systolic blood pressure (SBP) ≥140 mmHg, or diastolic blood pressure (DBP) ≥90 mmHg, or a previous diagnosis by a professional medical institution, or the use of antihypertensive medication in the past 2 weeks (33, 34).

Blood pressure measurements utilized the professional portable OMRON HEM-7136 BP monitor, manufactured by OMRON in Kyoto, Japan. Participants were advised to avoid smoking and not ingesting alcohol, tea, or coffee for at least 30 min prior to their appointment. Following a resting period of no less than 5 min, the right upper arm served as the site for two sequential blood pressure readings while the individual was seated. These readings were then averaged to ascertain the final blood pressure value.

### 2.4 Statistical analysis

The software Stata 17.0, R 4.4.0, and GraphPad Prism 9.5 were used for data processing and analysis. Continuous variables at baseline data are reported as mean ± standard deviation or median (interquartile range, IQR), while categorical variables are presented as percentages. Continuous variables with normal distributions were assessed using *t*-tests or Analysis of Variance (ANOVA), whereas those not normally distributed were examined through rank-sum tests. Categorical data assessment was conducted with chi-square tests. Missing data were imputed using multiple imputation. To assess the potential relationship between sleep quality, PA levels, and hypertension prevalence. A restrictive cubic spline (RCS) model was used to evaluate the dose-response relationship, with adjustments for potential confounding factors such as sex, age, educational attainment, marital status, smoking, alcohol ingestion, DDS, BMI, and total PA or PSQI score. Binary logistic regression analysis was used to explore the associations between various levels of PA, or sleep quality and the prevalence of hypertension. This study further conducted sensitivity analyses to verify the robustness of the results. Model 1 was adjusted for age and sex. Model 2 further adjusted for educational attainment, marital status, smoking, alcohol consumption, DDS, and BMI. The results are presented as odds ratios (OR) with 95% confidence intervals (95% CI), with a two-sided *P* values <0.05 were considered statistically significant.

## 3 Results

### 3.1 Participant characteristics

**Figure 1:**
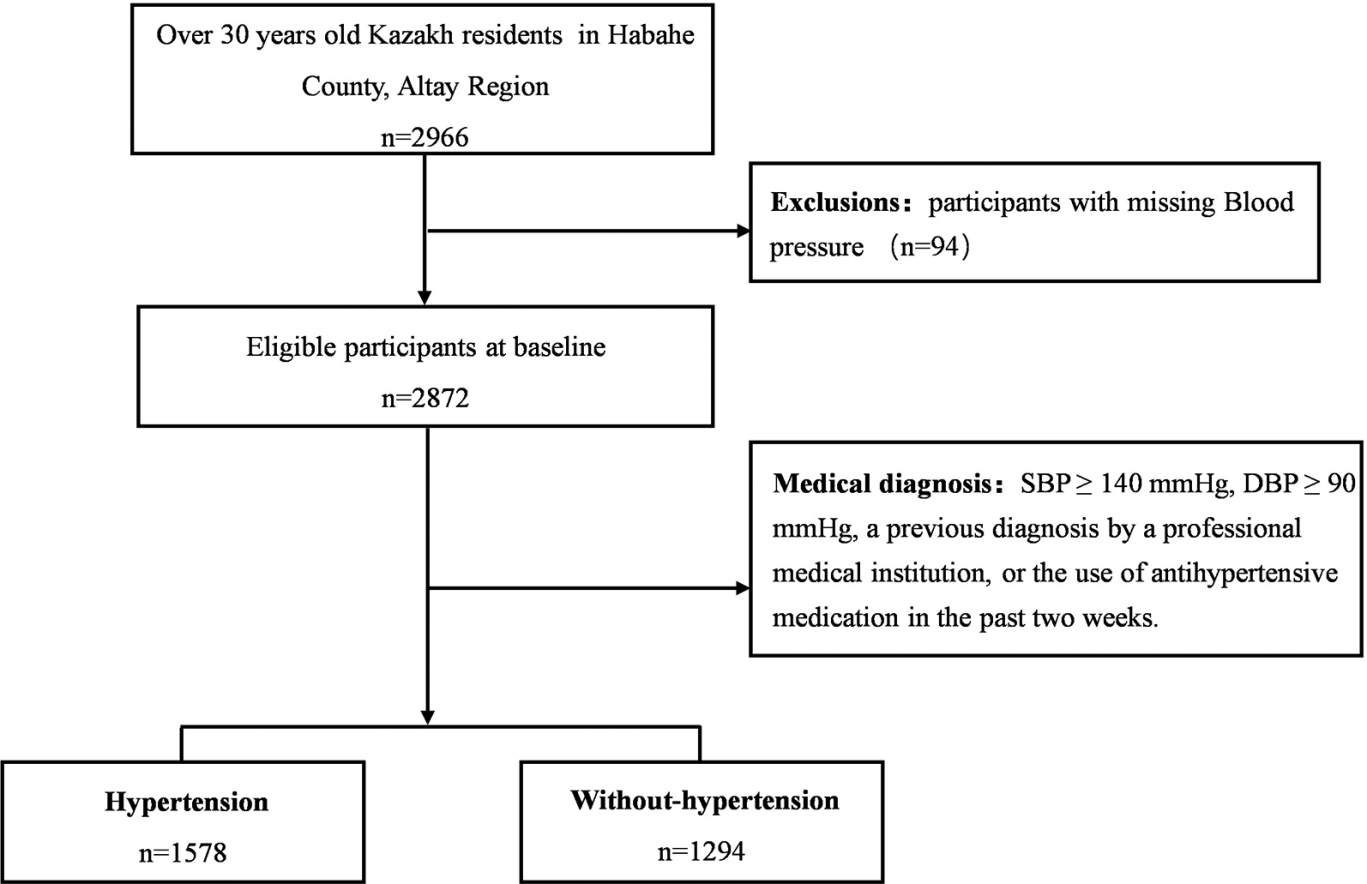
Flow chart of the participants of the present study.

In our study, a total of 2,966 participants aged 30 years or older were enrolled. After excluding individuals with missing valid blood pressure measurements (*n* = 94), the final analysis included 2,872 participants (Figure 1). The mean age of the participants was 50 ± 10 years. The median age was 49 (42–57) years, and 1,253 (43.63%) were male. Among the participants, 1,578 were diagnosed with hypertension, yielding a prevalence of 54.94%. Further analysis revealed that individuals with hypertension were older, had a higher BMI, lower educational attainment, and were more likely to have family history of hypertension compared to those non-hypertension. Additionally, hypertensive individuals had lower DDS, longer sedentary time, lower PA, and poorer sleep quality (all *P* < 0.05). However, no statistically significant differences were observed between the two groups in terms of household annual total income, alcohol ingestion, or tea-drinking habits (*P* > 0.05). Detailed sociodemographic, behavioral, and health characteristics are presented in Table 1.

**Table 1:** Baseline characteristics of participants.

Characteristics	Total (*n* = 2,872)	Hypertension (*n* = 1,578)	Non-hypertension (*n* = 1,294)	*P*-value
Age [years, median (IQR)]	49 (42, 57)	53 (47, 60)	44 (39, 51)	<0.001
Sex [male, *n* (%)]	1,253 (43.63)	789 (50.00)	464 (35.86)	<0.001
Educational attainment [below senior high school, *n* (%)]	2,105 (73.29)	1,192 (75.54)	913 (70.56)	0.003
Marital status [married, *n* (%)]	2,617 (91.12)	1,399 (88.66)	1,218 (94.13)	<0.001
Household annual total income [<60,000 CNY, *n* (%)]	1,888 (65.74)	1,044 (66.16)	844 (65.22)	0.599
BMI (*M* ± SD, kg/m−2)	28.95 ± 4.58	30.10 ± 4.65	27.56 ± 4.08	<0.001
Family history of hypertension [yes, *n* (%)]	1,871 (65.15)	1,073 (68.00)	798 (61.67)	<0.001
Smoking status [yes, *n* (%)]	613 (21.34)	387 (24.52)	226 (17.47)	<0.001
Alcohol ingestion [yes, *n* (%)]	695 (24.20)	379 (24.02)	316 (24.42)	0.802
Drinking tea [yes, *n* (%)]	2,671 (93.00)	1,471 (93.22)	1,200 (92.74)	0.613
DDS (score, *M* ± SD)	7.14 ± 1.30	7.04 ± 1.31	7.28 ± 1.29	<0.001
Sleep quality [score, median (IQR)]	4 (2,7)	4 (2,8)	3 (2,7)	0.004
Healthy	1,627 (56.65)	852 (53.99)	775 (59.89)	0.006*
Intermediate	564 (19.64)	329 (20.85)	235 (18.16)	
Poor	681 (23.71)	397 (25.16)	284 (21.95)	
Sedentary time [min/day, median (IQR)]	240 (120, 240)	240 (120, 300)	180 (120, 240)	<0.001
PA level [METs × min/week, median (IQR)]	6,132 (3,901.5, 9,198)	5,838 (3,306, 9,198)	8,358 (4,158, 9,198)	<0.001
Low	1,369 (47.67)	810 (51.33)	559 (43.20)	<0.001*
Moderate	1,244 (43.31)	628 (39.80)	616 (47.60)	
High	259 (9.02)	140 (8.87)	119 (9.20)	

*M*, mean; SD, standard deviation; IQR, interquartile range; BMI, body mass index; PA, physical activity; DDS, dietary diversity score.

* *P*-value for the comparison of sleep quality or PA level between the hypertension and non-hypertension groups.

### 3.2 Associations of PA level or sleep quality with the prevalence of hypertension

**Figure 2:**
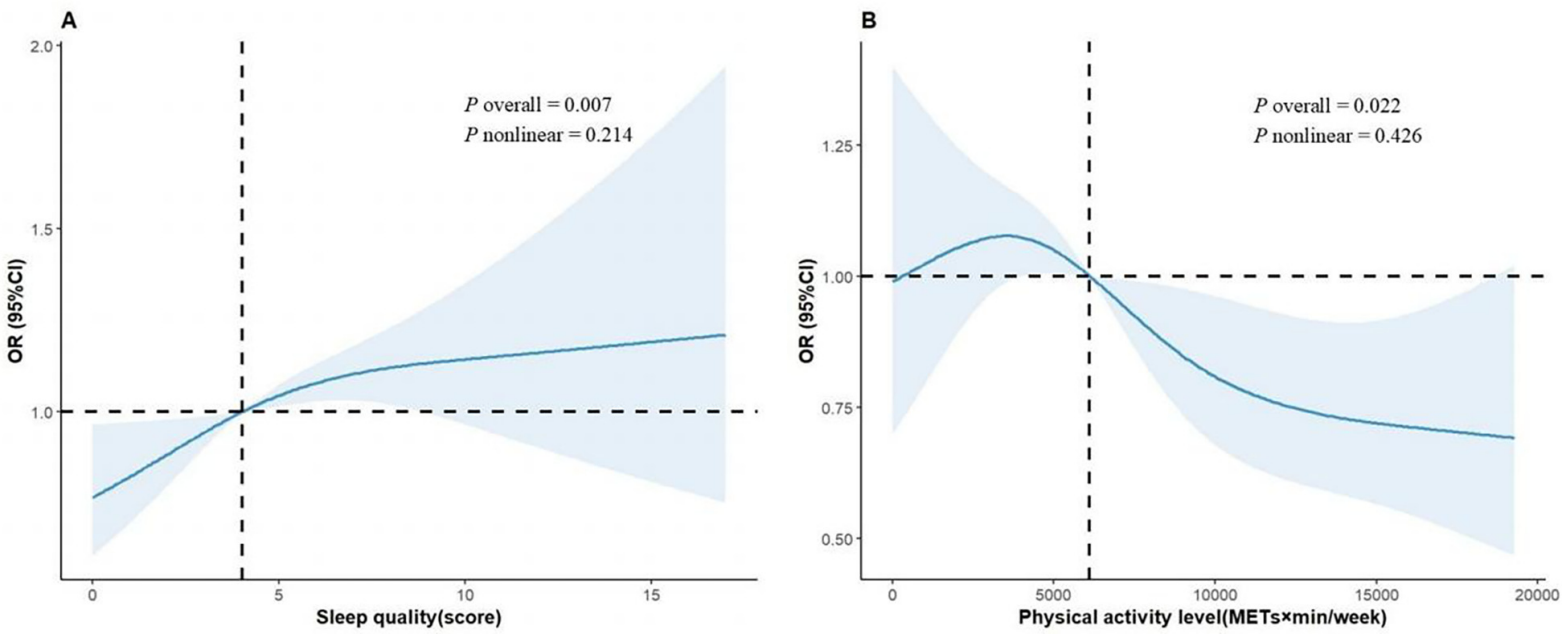
Dose-response relationship between sleep quality **(A)**, PA levels **(B)** and hypertension. In the RCS plot, sleep quality had three knots set at the 10th, 50th, and 90th percentiles of PSQI scores, while PA had four knots set at the 5th, 35th, 65th, and 95th percentiles of total PA. The solid line represents the point estimates; the shaded area represents the confidence intervals; and the black line is the reference line. Model was adjusted for age, sex, educational attainment, marital status, smoking status, household annual total income, alcohol ingestion, DDS, BMI, and PA or sleep quality.

As illustrated in Figure 2, the results of dose-response relationship indicate a significant positive association between sleep quality and the prevalence of hypertension (*P* overall = 0.002), suggesting that poor sleep quality may increase the prevalence of hypertension. However, the nonlinear trend was not statistically significant (*P* nonlinear = 0.130). Additionally, the association between PA level and hypertension prevalence was statistically significant (*P* overall = 0.022), while the nonlinear relationship remained nonsignificant (*P* nonlinear = 0.426). Notably, individuals with lower PA level (<6,000 METs × min/week) exhibited a relatively higher prevalence of hypertension. As PA level increased (>6,000 METs × min/week), the prevalence of hypertension gradually declined. However, at higher PA levels (>12,000 METs × min/week), the curve tended to plateau. This study further analyzed the effects of sleep quality and PA levels on the prevalence of hypertension in Table 2. Poor sleep quality was positively associated with the prevalence of hypertension. In Model 2, after adjusting for potential confounders, compared with individuals with healthy sleep quality, the prevalence of hypertension increased in the intermediate sleep quality group (OR = 1.33, 95% CI: 1.07–1.65, *P* = 0.011) and the poor group (OR = 1.38, 95% CI: 1.12–1.69, *P* = 0.001). However, compared with individuals with low PA level, the prevalence reduction in individuals with moderate PA and high PA level was no longer statistically significant (OR = 0.86, 95% CI: 0.72–1.02, *P* = 0.083; OR = 1.00, 95% CI: 0.74–1.34, *P* = 0.186). The trend test results showed that sleep quality was significantly positively correlated with hypertension prevalence (*P* for trend = 0.002), with the prevalence increasing as poorer sleep quality. However, the trend between PA level and hypertension prevalence was non-significant (*P* for trend = 0.186).

**Table 2:** Logistic regression analysis for the associations between PA level/sleep quality and the prevalence of hypertension.

Characteristic	*n*	Hypertension	Model 1			Model 2		
		*n* (%)	OR	95% CI	*P*-value	OR	95% CI	*P*-value
Sleep quality								
Health	1,627	852 (52.37)	ref			ref		
Intermediate	564	329 (58.33)	1.38	(1.12, 1.69)	0.002	1.33	(1.07, 1.65)	0.011
Poor	681	397 (58.30)	1.43	(1.17, 1.74)	<0.001	1.38	(1.12, 1.69)	0.002
*P* for trend					<0.001			0.002
PA level								
Low	1,369	810 (59.17)	ref			ref		
Mid	1,244	628 (50.48)	0.83	(0.70, 0.97)	0.022	0.86	(0.72, 1.02)	0.083
High	259	140 (54.05)	0.91	(0.69, 1.21)	0.533	1.00	(0.74, 1.34)	0.991
*P* for trend					0.071			0.186

Model 1 was adjusted for sex and age. Model 2 was adjusted for age, sex, educational attainment, marital status, smoking status, household annual total income, alcohol ingestion, DDS, BMI and sleep quality or PA.

**Figure 3:**
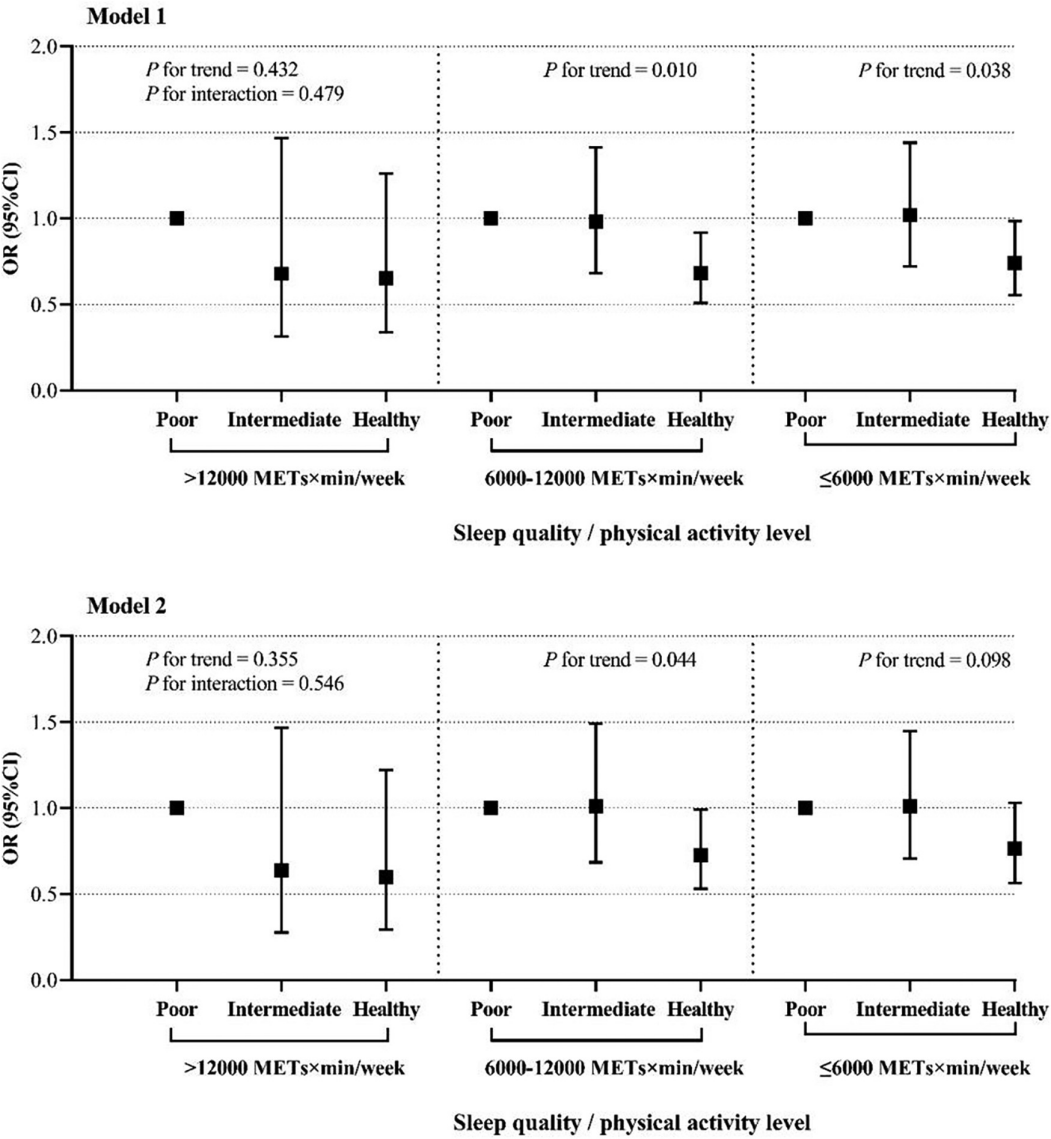
The relationship between sleep quality and the prevalence of hypertension at different PA levels. The figure presents odds ratios (ORs) and 95% confidence intervals (CIs) for hypertension prevalence across different sleep quality categories (poor, intermediate, and healthy) stratified by PA levels (>12,000 METs × min/week, 6,000–12,000 METs × min/week, and 56,000 METs × min/week). Model 1 was adjusted for age and sex. Model 2 was adjusted for age, sex, educational attainment, marital status, smoking status, household annual total income, alcohol ingestion, DDS, BMI. *P* for trend indicates the trend across sleep quality categories within each PA level group, and *P* for interaction assesses the interaction between sleep quality and PA level on hypertension prevalence.

Further analysis of the relationship between sleep quality and the prevalence of hypertension at different levels of PA is presented in Figure 3. In the moderate PA group, after adjusting for age and sex, individuals with healthy sleep quality had 32% lower risk of hypertension compared with those with poor sleep quality (OR = 0.68, 95% CI: 0.51–0.92, *P* = 0.011; *P* for trend = 0.006). This association remained statistically significant after additional adjustment for confounding factors (OR = 0.71, 95% CI: 0.52–0.97, *P* = 0.031), and trend analyses indicated that improved sleep quality might provide a protective effect against hypertension (*P* for trend <0.05). Further analysis was conducted to examine the relationship between sleep quality and hypertension prevalence (Figure 3). In the moderate-intensity physical activity (PA) group, after adjusting for age and sex, individuals with healthy sleep quality had a 32% lower risk of hypertension compared to those with poor sleep quality (OR = 0.68, 95% CI: 0.51–0.92, *P* = 0.011; *P* for trend = 0.006). This association remained statistically significant after further adjustment for potential confounders (OR = 0.71, 95% CI: 0.52–0.97, *P* = 0.031), suggesting that improving sleep quality may have a protective effect against hypertension in this population (*P* for trend <0.05). In the low physical activity group, where model 1 (adjusted for age and sex) showed that individuals with healthy sleep quality had a lower risk of hypertension (OR = 0.74, 95% CI: 0.55–0.98, *P* = 0.039), although this difference did not reach statistical significance in model 2 (OR = 0.76, 95% CI: 0.56–1.02, *P* = 0.076). Interaction analyses suggested that there was no significant interaction between PA level and sleep quality (*P* for interaction >0.05). Notably, in the high PA group, the association between sleep quality and hypertension prevalence did not also have statistical significance (*P* > 0.05).

### 3.3 Sensitivity analysis

To assess the robustness of our findings under different hypertension diagnostic criteria, we redefined hypertension by lowering the threshold from 140/90 to 130/80 mmHg and conducted a sensitivity analysis (Supplementary Tables S1, S2). The results demonstrated that the associations between sleep quality, PA levels, and hypertension prevalence remained consistent regardless of the diagnostic criteria applied. Even under the more stringent blood pressure threshold, sleep quality and PA continued to exert a significant influence on hypertension risk. These findings further reinforce the reliability and generalizability of our conclusions.

## 4 Discussion

To the best of the authors' knowledge, this is the first study to focus on agricultural workers on aged 30 and above in the remote regions of northwest China, aiming to investigate the impact of physical activity and sleep quality on the prevalence of hypertension among participants. The results indicated that the median PA level among participants was approximately 6,659 METs × min/week, which is significantly higher than the 3,213 METs × min/week observed in a previous study on Chinese adults (35). This elevated level of physical activity may be attributed to the participants’ rural backgrounds and their involvement in labor-intensive agricultural work. Such occupational activities are characterized by high intensity, long duration, and repetitive motions, typically utilizing only about 30% of maximal aerobic capacity, which does not lead to a sustained increase in blood pressure (36).

We observed a complex relationship between PA levels and hypertension prevalence. When PA exceeded 6,000 METs × min/week, a significant reduction in hypertension prevalence was observed. Previous research has confirmed that PA levels above the thresholds recommended by The WHO Guidelines on PA and sedentary behavior can significantly reduce the risk of cardiovascular diseases, including hypertension (17, 29). Proposed mechanisms include enhanced cardiovascular function, improved endothelial function, reduced systemic inflammation, better glycemic control, and weight management (19, 37). However, there remains controversy regarding whether excessive or high-intensity PA continues to confer health benefits (18, 35). In our study, the decreasing trend in hypertension prevalence plateaued beyond 12,000 METs × min/week, a finding consistent with prior research (35, 38), suggesting a potential “threshold effect”: while moderate exercise is beneficial for blood pressure control, excessive PA may induce oxidative stress and autonomic nervous system imbalance, partially offsetting its protective effects. Similar findings have been reported in studies examining PA and mortality risk (20, 36, 39). According to the “physical activity paradox”, different types of PA have different effects on health (40, 41). Previous research has predominantly focused on leisure-time physical activity (LTPA) and its significant health benefits (42). However, the PA observed in this study is predominantly occupational, particularly related to agricultural work, and its effects may differ from those associated with LTPA (43). LTPA primarily comprises dynamic activities of higher intensity and shorter durations, while occupational physical activity is characterized by repetitive, high-intensity activities, often involving continuous static muscle load and limited recovery time between high-intensity tasks, leading to increased physiological stress and potentially limiting cardiovascular benefits (41). In the study, less than 10% of the participants reported that PA exceeding 12,000 MET-min/week. Beyond this threshold, the negative impacts such as physiological strain, insufficient recovery, and occupational stressors may potentially overshadow the benefits associated with PA, compounding the relationship between occupational activity and hypertension. In contrast, sleep quality demonstrated a significant inverse association with hypertension prevalence. Compared to individuals with good sleep quality, those with intermediate and poor sleep quality had 33% and 38% higher risks of hypertension, respectively. This aligns with previous research (12, 44, 45), indicating that sleep disorders may elevate hypertension risk via mechanisms such as sympathetic nervous system activation, increased inflammation, and endothelial dysfunction (46, 47). A prospective cohort study based on UK Biobank data showed that maintaining healthy sleep patterns is associated with reduced hypertension incidence and fewer cardiovascular events in individuals already diagnosed with hypertension (2). Similarly, a longitudinal cohort study among younger and middle-aged adults supported the conclusion that poor sleep quality increases the risk of developing hypertension (48). Our findings provide additional empirical evidence for the relationship between sleep quality and hypertension risk.

Further stratified analysis revealed that, among participants with moderate PA, those with good sleep quality had significantly lower hypertension risk than those with poor sleep quality, suggesting that improving sleep may be particularly beneficial in this subgroup. Moreover, our study did not find a significant interaction between sleep quality and PA level in relation to hypertension prevalence. This differs from findings in some previous studies. For example, an Australian study on middle-aged women reported that the coexistence of sleep disturbances and insufficient PA significantly increased hypertension risk (49). Another study exploring the joint effects of PA and sleep duration on mortality risk suggested additive or multiplicative effects on cardiovascular and all-cause mortality (50). The lack of interaction observed in our study may be due to differences in study populations, assessment methods for PA and sleep, or statistical modeling approaches. Additionally, the observed differences in ORs across strata might be due to chance, given the non-significant interaction. Thus, further investigation in studies adequately powered to detect such interactions is required.

There are several limitations to this study. First, as a cross-sectional study, it is unable to establish causal relationships between sleep quality, PA levels, and hypertension. Future prospective cohort studies will be essential to validate our findings and provide clearer insights into the causal relationships. Second, the sample size of the high PA group was small, which may potentially bias the results and limit the generalizability of the findings for this specific subgroup. Third, questionnaire survey was used in the study, and there was a possibility of meeting bias when participants recalled related sleep and PA. Finally, although we accounted for numerous hypertension-related risk factors and adjusted for them in the analysis, the potential confounding from unmeasured variables cannot be completely ruled out. Despite these limitations, our study is well-designed, employs reliable methodology, and considers a comprehensive range of factors, providing valuable empirical support for the prevention and management of hypertension among agricultural workers.

PA and sleep quality are associated with the prevalence of hypertension. Among individuals with moderate levels of PA, poor sleep quality is significantly linked to an increased risk of hypertension. Our findings underscore the importance of implementing health interventions that target both PA and sleep quality. Future research should incorporate objective devices to assess the health impacts of both sleep and PA, and conduct prospective studies focused on these behaviors.

## Data Availability

The raw data supporting the conclusions of this article will be made available by the authors, without undue reservation.
