# Supplementary material for: Joint association of sleep quality and physical activity with hypertension: a cross-sectional population study in agricultural workers
Source: Front Cardiovasc Med. 2025 Aug 11;12:1618094. doi: 10.3389/fcvm.2025.1618094 (PMC12375635; doi:10.3389/fcvm.2025.1618094)
Supplement: Supplementary file 1 [file Table1.docx]

**Supplementary Materials**

**Supplementary Table 1. Logistic regression analysis for the associations between PA level/sleep quality and the prevalence of hypertension among participants diagnosed at 130/80 mmHg**

| Characteristic | Model 1 | | | Model 2 | | |
| --- | --- | --- | --- | --- | --- | --- |
|  | OR | 95%CI | *P* | OR | 95%CI | *P* |
| Sleep quality |  |  |  |  |  |  |
| Health | ref |  |  | ref |  |  |
| Intermediate | 1.34 | (1.06,1.68) | 0.014 | 1.29 | (1.01,1.64) | 0.041 |
| Poor | 1.59 | (1.28,1.99) | <0.001 | 1.55 | (1.23,1.95) | <0.001 |
| *P* for trend |  |  | <0.001 |  |  | <0.001 |
| PA level |  |  |  |  |  |  |
| Low | ref |  |  | ref |  |  |
| Mid | 0.81 | (0.67,0.97) | 0.023 | 0.82 | (0.68,1.00) | 0.050 |
| High | 0.89 | (0.64,1.22) | 0.461 | 0.89 | (0.63,1.25) | 0.500 |
| *P* for trend |  |  | 0.076 |  |  | 0.148 |

Model 1 was adjusted for sex, age. Model 2 was adjusted for age, sex, educational attainment, marital status, smoking status, household annual total income, alcohol ingestion, DDS, BMI and sleep quality or PA.

**Supplementary Table 2 The relationship between sleep quality and hypertension prevalence among participants diagnosed at 130/80 mmHg at different PA levels**

| PA level | Sleep quality | Model 1 | | | | Model 2 | | | |
| --- | --- | --- | --- | --- | --- | --- | --- | --- | --- |
|  |  | OR | 95%CI | *P* for trend | *P* for interaction | OR | 95%CI | *P* for trend | *P* for interaction |
|  | Poor | ref |  |  |  | ref |  |  |  |
| Low | Intermediate | 0.91 | (0.60,1.38) | 0.284 | 0.084 | 0.93 | (0.62,1.38) | 0.083 | 0.091 |
|  | Health | 0.77 | (0.54,1.08) |  |  | 0.71 | (0.51,0.99) |  |  |
|  |  |  |  |  |  |  |  |  |  |
| Moderate | Poor | ref |  |  |  | ref |  |  |  |
|  | Intermediate | 0.84 | (0.54,1.29) | 0.013 |  | 0.82 | (0.54,1.24) | 0.006 |  |
|  | Health | 0.61 | (0.43,0.87) |  |  | 0.6 | (0.43,0.84) |  |  |
|  |  |  |  |  |  |  |  |  |  |
| High | Poor | ref |  |  |  | ref |  |  |  |
|  | Intermediate | 0.52 | (0.19,1.41) | 0.165 |  | 0.54 | (0.21,1.39) | 0.133 |  |
|  | Health | 0.43 | (0.18,1.03) |  |  | 0.43 | (0.19,0.98) |  |  |

Model 1 was adjusted for sex, age. Model 2 was adjusted for age, sex, educational attainment, marital status, smoking status, household annual total income, alcohol ingestion, DDS, and BMI.
